# Neck of Femur Fractures in the First Eight Months of the COVID-19 Pandemic: A UK Systematic Review and Meta-Analysis

**DOI:** 10.7759/cureus.20262

**Published:** 2021-12-08

**Authors:** Loukas Andritsos, Owain Thomas, Susil Pallikadavath, Sayyied Kirmani, Sharan Sambwhani

**Affiliations:** 1 Trauma and Orthopaedics, Northampton General Hospital, Northampton, GBR; 2 Cardiology, Leicester General Hospital, Leicester, GBR; 3 Trauma and Orthopaedics, Kettering General Hospital, Kettering, GBR

**Keywords:** hip and proximal femur trauma, coronavirus disease 2019, time to surgery, orthopaedic research, mortality rates, neck of femur fracture, covid-19

## Abstract

Neck of femur (NOF) fracture patients have significant 30-day mortality. The incidence of NOF fractures remained high during the coronavirus disease 2019 (COVID-19) pandemic in the United Kingdom. Consequently, numerous cases were complicated with concurrent severe acute respiratory syndrome coronavirus 2 infection. We performed a systematic review and meta-analysis of all studies from the United Kingdom related to NOF fractures and 30-day mortality outcomes during the pandemic. A systematic review and meta-analysis was performed and reported as per the Preferred Reporting Items for Systematic reviews and Meta-Analyses guidelines. Two reviewers independently searched on Medline for studies that were published between the 1st of March 2020 and the 1st of November 2020 in the United Kingdom. The following outcomes were compared: 30-day mortality, time to surgery, and anaesthetic risk. A total of five articles were included in this review. In total, 286 patients with NOF fractures and COVID-19 infection were identified, with 30-day mortality ranging from 30.5% to 50% (odds ratio = 6.02; 95% confidence interval = 4.10-8.85; χ^2^ = 4.82; I^2^ = 58%). Increased time to surgery due to COVID-19-related delays was also noted for the majority of patients in some studies. Mortality scores (Charlson Comorbidity Index, Nottingham Hip Fracture Score) failed to accurately predict the mortality risk. Concurrent infection of COVID-19 in patients with NOF fractures increases the 30-day mortality sixfold compared to the COVID-19-negative group. Efforts should be made to optimise time to surgery as well as consideration of postoperative care in higher dependency units. Future updates in mortality predicting scores should include COVID-19 infection as a significant factor.

## Introduction and background

The coronavirus disease 2019 (COVID-19) outbreak, caused by the novel severe acute respiratory syndrome coronavirus 2 (SARS-CoV-2), has caused over 130,000 deaths in the United Kingdom (UK). Although the disease can affect individuals of all ages, patients above the age of 65 have significantly higher mortality, accounting for 89.5% of all COVID-19-related deaths in the UK [1].

Orthopaedic centres in the UK have experienced a significant reduction in trauma cases during the national lockdown; however, the number of patients sustaining neck of femur (NOF) fractures have remained relatively stable [2]. NOF fractures are known to be a significant cause of morbidity and mortality worldwide [3]. In 2019, the 30-day mortality published by the National Hip Fracture Database for proximal femur fracture patients in England was 6.5% [4]. Since the onset of the pandemic, several studies have analysed the outcomes of hip fractures in patients with and without COVID-19. The COVIDSurg [5] collaborative found that pulmonary complications occur in approximately half of the patients undergoing surgery with concurrent COVID-19 infection. Despite these results, the importance of continuing operative management for NOF patients during the pandemic was highlighted by Mi et al. [6], who found that delayed surgery had better outcomes and fewer complications than non-operative management.

In this study, we performed a systematic review and meta-analysis of studies published in the UK over the first eight months of the pandemic to delineate the impact of COVID-19 on 30-day mortality outcomes of patients with NOF fractures.

## Review

Methodology

Ethical approval for this study was deemed unnecessary because it was a review of existing literature and did not involve any handling of individual patient data.

Two reviewers independently searched for studies published between the 1st of March 2020 and the 1st of November 2020 on Medline. Search keywords included “coronavirus,” “COVID-19,” “SARS-COV-2,” and “trauma and orthopaedics,” or “trauma and orthopedics” and “trauma,” “fracture,” “trauma *NOT mental.” Further articles were manually reviewed using the reference lists of identified articles.

Eligibility criteria for our study included any randomised controlled trials and/or case series of patients over 60 years of age with a diagnosis of NOF fracture complicated by COVID-19 infection. For inclusion in the review, studies had to present results on 30-day mortality as the primary outcome and American Society of Anesthesiology (ASA) score, Nottingham Hip Fracture Score (NHFS), or time to surgery as secondary outcomes. Only studies reported from the UK were included. The type of fracture had to be intracapsular, peritrochanteric, or subtrochanteric.

Studies were excluded if they were conducted outside of the UK, or if they included patients with midshaft femoral, distal femur, or periprosthetic fractures.

Two reviewers independently assessed abstracts and full-texts for suitability (LA and OT). Disagreements were resolved by a third reviewer (SS). Data were extracted by one investigator (LA) and checked by a second reviewer (OT). The following data were extracted: first author’s name, type of study, cohort size, demographics, perioperative mortality scores, type of surgery, and postoperative mortality. The primary outcome of interest was 30-day mortality and secondary outcomes were the time to surgery and perioperative mortality scores.

Data analysis

Revman 5.4 was used for data analysis. A random-effects model using the Mantel-Haenszel method was used as heterogeneity was expected to be high [7]. Pooled relative risk (RR), pooled risk difference, and the number needed to treat or harm (NNT/NNH) were calculated.

Results

**Figure 1:**
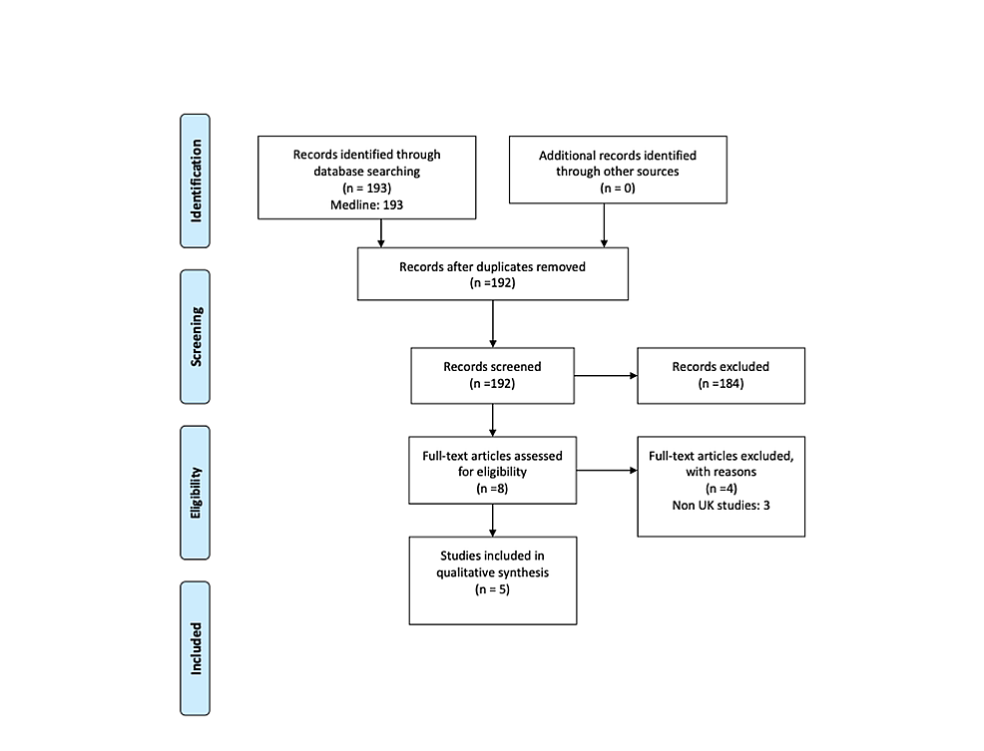
PRISMA flow diagram. PRISMA: Preferred Reporting Items for Systematic reviews and Meta-Analyses

The systematic search of Medline identified 193 potentially eligible studies. We found no additional records during manual searches of reference lists. Figure 1 presents the Preferred Reporting Items for Systematic reviews and Meta-Analyses (PRISMA) flow diagram. We removed one study as duplicate and 184 studies based on their title and abstract descriptions. Full texts from eight studies were obtained and read, of which three were excluded as they were non-UK-based. Finally, five studies were included in this systematic review.

Of the five studies, four [8-11] were retrospective and one [12] was a prospective cohort case series. These studies were all conducted in the UK, were published between 1 March 2020 and 1 November 2020, and included 222 cases of NOF fracture patients with COVID-19 infection.

The five studies included a total of 1,422 patients with proximal femur fractures, of whom 286 tested positive for COVID-19. The mean age was similar for four out of five studies, ranging between 83 and 86 years. One study [10] had a mean age of 71.9. Table 1 presents the study type, number of patients, and 30-day mortality for all studies included in this systematic review.

**Table 1:** Study type, number of patients, and 30-day mortality of studies included in the systematic review. COVID-19: coronavirus disease 2019; NOF: neck of femur fracture; NHFS: Nottingham Hip Fracture Score; CCI: Charlson Comorbidity Index; T: Time to theatre; M: 30-day mortality

Study	Type of study	Number of COVID-19-positive NOF fracture patients	Outcome assessments	30-day mortality of COVID-19-positive patients
Narang et al. [12]	Prospective multicentre cohort	86	NHFS, T, M	34.9%
Dupley et al. [11]	Retrospective multicentre cohort	64	CCI, M, T	32.8%
Kayani et al. [10]	Retrospective multicentre cohort	82	M, T	30.5%
De et al. [9]	Retrospective multicentre cohort	34	CCI, T, M	41.2%
Fadulelmola et al. [8]	Retrospective multicentre cohort	20	NHFS, CCI, T, M	50

Thirty-Day Mortality

All five studies reviewed 30-day mortality for COVID-19-positive patients with NOF fractures. Each study found significantly higher mortality in the COVID-19-positive group compared to the national average, ranging between 30.4% and 50%. Overall, the average 30-day mortality from all studies was 37.88%. Some of the studies reported the time to theatre, comorbidity prognostic scores such as Charlson Comorbidity Index (CCI) or Nottingham Hip Fracture Score (NHFS). In addition, the studies compared the mortality of COVID-19-positive patients to either COVID-19-negative patients from the same time period or current mortality figures documented nationally.

Two studies [9,11] investigated COVID-19-positive patients with NOF fractures and analysed attributes within the group to identify possible contributing factors to higher mortality. De et al. [9] reported a 30-day mortality of 41.2% in the COVID-19-positive group. Moreover, the presence of respiratory symptoms and/or changes in the postoperative chest X-ray were the only two factors with a statistically significant impact on the overall mortality. Dupley et al. [11] reported a 30-day mortality of 32.8%. In their study, the presence of myocardial infarction in the patient’s medical history was the only factor affecting 30-day mortality (p < 0.05).

Three studies [8,10,12] compared 30-day mortality between COVID-19-positive and negative patients sustaining a NOF fracture over the same time period. Narang et al. [12] performed a prospective cohort study. They presented the findings of 682 NOF fracture patients, including 86 COVID-19-positive patients. The overall mortality in the COVID-19-positive group was 34.9% versus 6% in the COVID-19-negative group. RR was 3.00 for COVID-19-positive patients.

Fadulemola et al. [8] analysed a relatively small sample (75 NOFs, 20 positive for COVID-19) and reported a mortality rate of 50% in the group testing positive for COVID-19. The prospective study by Narang et al. [12] had the largest sample size (682 NOF patients, 86 [12.6%] of whom tested positive for COVID-19) and reported a mortality rate of 34.9% in the COVID-19-positive group. Finally, Kayani et al. [10] performed a large multicentre retrospective study with 82 COVID-19-positive patients showing mortality of 30.5% versus 10.3% in COVID-19-negative patients (35/340). Table 2 presents the combined 30-day mortality in NOF femur fractures with and without concurrent COVID-19 infection reported in the included studies.

**Table 2:** Combined 30-day mortality in NOF femur fractures with and without concurrent COVID-19 infection in included studies. COVID-19: coronavirus disease 2019; NOF: neck of femur fracture

	Mean age of COVID-19-positive patients	Number of COVID-19-positive NOF fracture patients	30-day mortality of COVID-19-positive patients (%)	Number of COVID-19-negative NOF fracture patients	30-day mortality of COVID-19-negative patients (%)
Valid	5	5	5	3	3
Missing	0	0	0	2	2
Mean	82.10	57.20	37.88	330.33	7.86
SD	5.85	29.21	7.85	270.63	2.20
Minimum	71.90	20.00	30.50	55.00	6.00
Maximum	86.00	86.00	50.00	596.00	10.30
Overall		286.00	37.88	991.00	7.86

Time to Surgery

Three studies [8,9,12] analysed the time to surgery to assess its potential contribution to mortality. As per the National Institute for Health and Care Excellence guidance on hip fractures [13], operative delay has been associated with several adverse outcomes, including increased length of stay, pressure ulcers, and decreased return to independent living. This is reflected by the Best Practice Tariff Initiative for hip fractures which rewards surgery within 36 hours.

Narang et al. [12] reported that 22.1% of patients in the COVID-19-positive group breached the 36 hours versus 40.8% in the COVID-19-negative group. The time to surgery was 72 hours (median) for both COVID-19-positive and negative groups in the study by Kayani et al. [10]. This did not show statistically significant differences in mortality between the two groups. De et al. [9] compared the time to surgery for COVID-19-positive patients who survived with those who did not and showed showing statistically non-significant difference (mean duration of 46.7 hours vs. 54.1 hours, respectively). Dupley et al. [11] showed that 67% of COVID-19-positive patients achieved the 36-hour tariff. Lastly, Fadulelmola et al. [8] reported a mean duration of 37.4 hours from admission to surgery.

Morbidity Scores

In total, four studies reported CCI and/or NHFS scores. Dupley et al. [11] found no difference in CCI scores between patients who died and those who survived with a mean score of 6 in each category. Fadulelmola et al. [8] found a mean NHFS of 6 and CCI of 5.4 in their study including 20 COVID-19-positive NOF fracture patients. Moreover, these scores were only marginally higher from the COVID-19-negative group (5.5 and 5.1, respectively). Narang et al. [12] found a statistically significant difference in NHFS scores between the COVID-19-positive and negative groups (5.9 and 5.0, respectively), showing a higher score for the positive group. De et al. [9] reported CCI and NHFS scores of 5.5 and 6, respectively, in a group of 34 COVID-19-positive patients. The 14 patients who died within 30 days had higher scores compared to the survivor group; however, this was not statistically significant. Table 3 presents the time to surgery and morbidity scores reported in the studies included in this review.

**Table 3:** Time to surgery and morbidity scores for selected studies. COVID-19: coronavirus disease 2019; NOF: neck of femur

Study	Time to surgery for COVID-19-positive NOF patients (hours)	Time to surgery for COVID-19-positive patients <36 hours (%)	Charlson Comorbidity Index	Nottingham Hip Fracture Score
Narang et al. [12]	N/A	77.9	N/A	5.0
Dupley et al. [11]	N/A	67	6	N/A
Kayani et al. [10]	72	N/A	N/A	N/A
De et al. [9]	49.6	N/A	5.5	6
Fadulelmola et al. [8]	37.4	N/A	5.4	6

Risk Factors: Comorbidities

**Figure 2:**
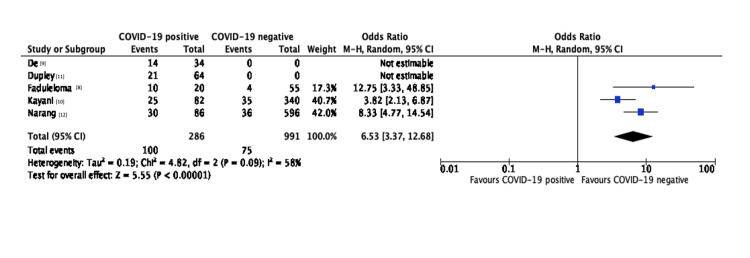
Results of the meta-analysis. COVID-19: coronavirus disease 2019; CI: confidence interval

Overall, three studies reported on other independent factors that may have affected the mortality of COVID-19-positive patients. Kayani et al. [10] found that smoking and the presence of more than three comorbidities were statistically significant, increasing the risk of mortality in the COVID-19-positive group (82 patients) but not in the COVID-19-negative group (340 patients). Fadulelmola et al. [8] found that C-reactive protein and white cell count were higher in the COVID-19 group. Lastly, Dupley et al. [11] showed that a history of myocardial infarction had a statistically significant impact on mortality in COVID-19-positive patients (9% incidence in the survivor group vs. 33% in the mortality group). Figure 2 presents the overall results of the meta-analysis.

Discussion

The statistics of the National Hip Fracture Database showed an average 30-day mortality rate of 6.5% for 2019 [4]. Compared to the 37.88% overall 30-day mortality from the five UK studies included in this meta-analysis, we can see the suspected effect of COVID-19 on this vulnerable population. Findings from a New York [14] and a Spanish study [15] are also consistent with the UK results for the same time period, although these studies included a smaller sample size. Egol et al. [14] showed a 30-day mortality rate of 53% in a group of 17 patients with NOF fracture, whereas Vives et al. [15] showed a 30-day mortality rate of 30.4% in 23 COVID-19-positive patients with NOF fracture.

Time to surgery did not show any statistically significant impact on mortality in the five studies included in this meta-analysis. Similar findings were reported by Vivez et al. [15] who showed no relationship between the survival and time to surgery. This was not the case for the study by Egol et al. who found that the COVID-19-positive patient group had statistically significant increased time from admission to surgery compared to the COVID-19-negative group (2.7 [SD 3] days vs. 1.1 [SD 0.6]). Interestingly, the COVID-19-negative group from the latter study [15] showed that these patients were taken to the theatre in 1.1 days on average with a narrow SD of 0.6 days. It is possible that prompt time to surgery contributed to the good overall outcome for the latter group. However, in the COVID-19-positive group, it is possible that patients were not medically optimized to undergo surgery. Additionally, the delayed surgery time can be attributed to departmental understaffing of healthcare professionals, due to personal health or self-isolation, seen in all sectors during the pandemic. Furthermore, theatre list space may have been reduced during lockdown due to staff unavailability due to sickness or redeployment in medical or intensive care posts. With hospitals now having adjusted to the pandemic, further studies would provide more insight into the impact of time to surgery in COVID-19-positive NOF fractures.

A study by Kang et al. [16] reviewed articles from several countries and reported age-specific COVID-19 mortality. The authors included studies from China, Korea, Italy [17], and Spain and showed a range in mortality in people over the age of 80 (14.8-26.6%). These were COVID-19-positive patients without NOF fracture. We can speculate from this difference in mortality that NOF fracture in combination with COVID-19 adds to the overall mortality rate of an elderly patient.

We also found that NHFS had a poor correlation with predicted mortality and overall outcomes. The studies that measured the NHFS [8,9,12] found scores between 5 and 6 for the COVID-19-positive groups, which predicted 30-day mortality at 4.6-7.4%. However, the actual 30-day mortality for these studies was 34.9%, 50%, and 41.2%, respectively.

An important note is that our review was performed before the vaccination programme began and only included unvaccinated individuals. The high mortality rate depicted in this review will continue to be the case for unvaccinated patients with NOF fracture. The implementation of the vaccination programme may significantly affect 30-day mortality, and further investigations are recommended.

## Conclusions

COVID-19 significantly increases mortality among patients with concurrent NOF fractures. The studies included in this systematic review precede the rollout of the UK COVID-19 vaccination programme. Studies on the mortality of vaccinated patients with NOF fractures and COVID-19 to see the overall benefit of the vaccines on this vulnerable population are recommended. As hospitals adapt to the pandemic and its implication on operative care, delays in surgery due to COVID-19 infection should reduce.

Current mortality scores do not accurately reflect 30-day mortality among COVID-19-positive patients. Concurrent infection in unvaccinated patients increases 30-day mortality approximately sixfold. Therefore, we advocate a modification of the NHFS/CCI scores to accommodate for the overall considerable impact of COVID-19 in unvaccinated patients with NOF fracture.
